# Fracture-Dedicated Prosthesis Promotes the Healing Rate of Greater Tuberosity in Reverse Shoulder Arthroplasty: A Meta-Analysis

**DOI:** 10.3389/fsurg.2021.616104

**Published:** 2021-12-09

**Authors:** Shu-Kun He, Jing-Ping Liao, Jin-Hai Guo, Fu-guo Huang

**Affiliations:** ^1^Department of Orthopedics, The First Affiliated Hospital, College of Medicine, Zhejiang University, Hangzhou, China; ^2^School of Nursing, Peking University, Beijing, China; ^3^Institute of Mental Health, The Sixth Hospital, Peking University, Beijing, China; ^4^National Clinical Research Center for Mental Disorders & Key Laboratory of Mental Health, Ministry of Health, Chinese Academy of Medical Sciences Research Unit (No. 2018RU006), Peking University, Beijing, China; ^5^Department of Orthopedics, West China Hospital, Sichuan University, Chengdu, China

**Keywords:** proximal humeral fractures, reverse shoulder arthroplasty, greater tuberosity, design of prosthesis, meta-analysis

## Abstract

**Introduction:** Reverse shoulder arthroplasty (RSA) is becoming popular in the treatment of complex proximal humeral fractures (PHFs). Greater tuberosity healing may influence functional outcomes and range of motion (ROM) of shoulder after RSA. In addition, the design of prosthesis may impact the healing rate of greater tuberosity. The purpose of this study is to know: (1) does the healing of greater tuberosity affect the functional outcomes and ROM of shoulder? and (2) does the design of prosthesis affect the healing rate of greater tuberosity?

**Materials and Methods:** PubMed, Ovid/Embase, and the Cochrane Library were searched for studies comparing the clinical outcomes between the healed groups and the non-healed groups after RSA.

**Results:** For functional outcomes, the results showed that the healed group had better Constant scores (CSs) (*p* < 0.0001). For ROM, the healed group showed better flexion (*p* < 0.0001), abduction (*p* = 0.02), and external rotation (*p* < 0.00001) of shoulder. For the design of prosthesis, the mean healing rate of greater tuberosity (82.7%) in patients with fracture-dedicated prosthesis was higher than those (63.0%) in patients with standard prosthesis. Subgroup analyses showed that the CS (*p* = 0.12) and abduction (*p* = 0.96) of patients using fracture-dedicated prostheses were not different between the healed groups and the non-healed groups. Meta-regression showed that there was no significant relationship between the design of prosthesis and CS (*p* = 0.312), flexion (*p* = 0.422), or external rotation (*p* = 0.776).

**Conclusion:** Our meta-analysis showed that the healed groups could obtain better functional outcomes and ROM than the non-healed groups. In addition, fracture-dedicated prostheses promoted the healing rate of greater tuberosity.

**Systematic Review Registration:** https://www.crd.york.ac.uk/prospero/display_record.php?ID=CRD42020157276, PROSPERO: CRD42020157276.

## Introduction

The incidence of proximal humeral fractures (PHFs) is about 5.7% of all the fractures in adults (1). For elderly patients, PHFs are the third most commonly fracture and account for 10% of all the fractures (2, 3). Currently, clinical treatment includes conservative treatment, fracture fixation, and arthroplasty. However, the optimal management of PHF in elderly patients remains challenging.

Reconstruction is often limited or impossible in elderly patients with PHF due to the osteonecrosis of humeral head, the degenerative changes of rotator cuff, or the high prevalence of osteoporosis (4, 5). Therefore, elderly patients could be treated with hemiarthroplasty (HA) or reverse shoulder arthroplasty (RSA). HA was a standard treatment for complex PHF previously, but functional outcomes were variable (6). Considering that clinical outcomes of HA highly depend on the anatomic healing of tuberosities, RSA is becoming popular in the treatment of complex PHF, as it relies mainly on deltoid muscle function (7–9).

The greater tuberosity was the major bony landmarks of the proximal humerus and it served as attachment points for the rotator cuff (10, 11). For early use of RSA in complex PHF, surgeons did not routinely reattach tuberosities and they found no differences in terms of functional outcomes and range of motion (ROM) of the shoulder between the healed greater tuberosity groups and the non-healed greater tuberosity groups (12–14). Some studies observed that elderly patients with the healed tuberosities after RSA showed improved active forward flexion, external rotation, and external rotation strength (15–17). In addition, a meta-analysis of seven studies showed that the healed tuberosity group achieved the higher Constant scores (CSs) and better forward flexion, abduction, and external rotation than the non-healed tuberosity group (18). Recently, Simovitch et al. reported that the healing of greater tuberosity significantly influenced external rotation of the shoulder, but they found that there was no difference in clinical outcomes between the healed group and the non-healed group (19). In addition, Reuther et al. also could not confirm that patients with the healed tuberosities would have better postoperative ROM than those with the non-healed tuberosities (20). The reason for the different observations may be due to the use of different designs of prostheses (fracture-dedicated prosthesis and standard prosthesis) in RSA.

Currently, a fracture-dedicated prosthesis could improve the healing rate of the tuberosity after HA compared with a standard prosthesis, which was always bulky and lacked the necessary fenestrations for bone graft and tuberosity suture fixation (21). In addition, Jeong et al. also reported that the non-healing rate of the tuberosity was higher in elderly patients with a standard prosthesis after RSA (22). Thus, the purpose of our meta-analysis study was: (1) to compare clinical outcomes of elderly patients after RSA between the healed greater tuberosity groups and the non-healed greater tuberosity groups and (2) to know whether the design of a prosthesis could affect the healing rate of the greater tuberosity.

## Methods

This systematic review and meta-analysis were conducted according to the Preferred Reporting Items for Systematic Reviews and Meta-Analyses (PRISMA) guidelines (23) and the Assessing the Methodological Quality of Systematic Reviews (AMSTAR) guidelines (24). We included studies with patients meeting the following criteria: (1) study design including randomized controlled trials (RCTs), cohort studies, and case–control studies; (2) adults greater than 55 years of age; (3) acute proximal humeral fracture; (4) treatment with reverse shoulder replacement; and (5) a minimum follow-up of 6 months. Studies that did not directly compare outcomes between the healing greater tuberosity group and non-healing greater tuberosity group were excluded. Chronic injuries, biomechanical, case reports, conference abstracts, and animal studies were excluded.

### Search Strategy

PubMed (up to November 2019), Ovid/Embase (up to November 2019), and the Cochrane Library (up to November 2019) were searched for articles published in English and other languages. The specific search strategies for all the databases are shown in Supplementary Material. We also checked the reference lists of identified relevant articles for additional relevant studies.

### Study Selection

Titles and abstracts of all the collected articles were screened independently by two authors. The full text of potentially eligible articles was obtained and assessed independently by both the authors using the predetermined inclusion and exclusion criteria. Any disagreements were resolved with consensus among all the authors.

### Data Extraction

Two review authors separately extracted data and discrepancies were resolved by discussion to reach a consensus. Extracted information included demographics of the patient, classification of fractures, implant types, follow-up time, and all the outcomes of interest. Outcomes of interest included functional scores, the ROM of shoulder, healing rates of greater tuberosity, and complications. Functional scores included the CS, the American Shoulder and Elbow Surgeons (ASES) score, the Disabilities of the Arm, Shoulder and Hand (DASH) questionnaire, the Subjective Shoulder Value (SSV), the Simple Shoulder Test (SST) score, and the Visual Analog Scale (VAS). The ROM of shoulder included flexion, abduction, external rotation, and internal rotation. Complications included implant-related complications, infections, nerve injuries, and so on.

### Quality Assessment

Two authors independently assessed the risk of bias in included studies and disagreements were resolved by discussion. The methodological quality of case–control studies and cohort studies was assessed using the Newcastle–Ottawa Scale (NOS) (25) and RCTs were assessed by the Cochrane Risk of Bias Tool (26).

### Statistical Analysis

Weight mean differences (MDs) were calculated for continuous outcomes with 95% CIs and two-sided *p*-values (27, 28). The results were pooled using a random-effects model due to differences in clinical or methodological characteristics of the included studies. When it is not explicitly expressed, SDs were estimated by mean and range of the study. For more than two subgroups in an included study (such as the healed group, the non-healed group, and the excision group), the mean and SD of subgroups (such as the non-healed group and the excision group) were combined through the merging method for further analysis (29). Heterogeneity was assessed using the chi-squared test and the *I*^2^ statistic was applied to these summary data to describe the percentage of variation across studies (30). A value of *I*^2^ more than 50 was considered as high heterogeneity (30). Subgroup analyses and meta-regression were performed to explore the sources of heterogeneity when substantial heterogeneity was present. Sensitivity analysis was carried out using the leave one-out approach to investigate the influence of an individual study on the pooled estimate. For meta-regression, the predefined covariates included the design of prosthesis and the origin country of studies. Potential publication bias was evaluated by the Begg's test (31) and the Egger's test (32). The trim-and-fill method was used to further assess the effect of publication bias (33). All the meta-analyses were performed using Review Manager software (RevMan version 5.3, Cochrane Collaboration, Copenhagen, Denmark). Sensitivity analyses, meta-regression, and publication bias were performed with Stata software, version 15 (StataCorp, College Station, Texas, USA). A *p*-value of 0.05 was considered as statistically significant.

## Results

**Figure 1:**
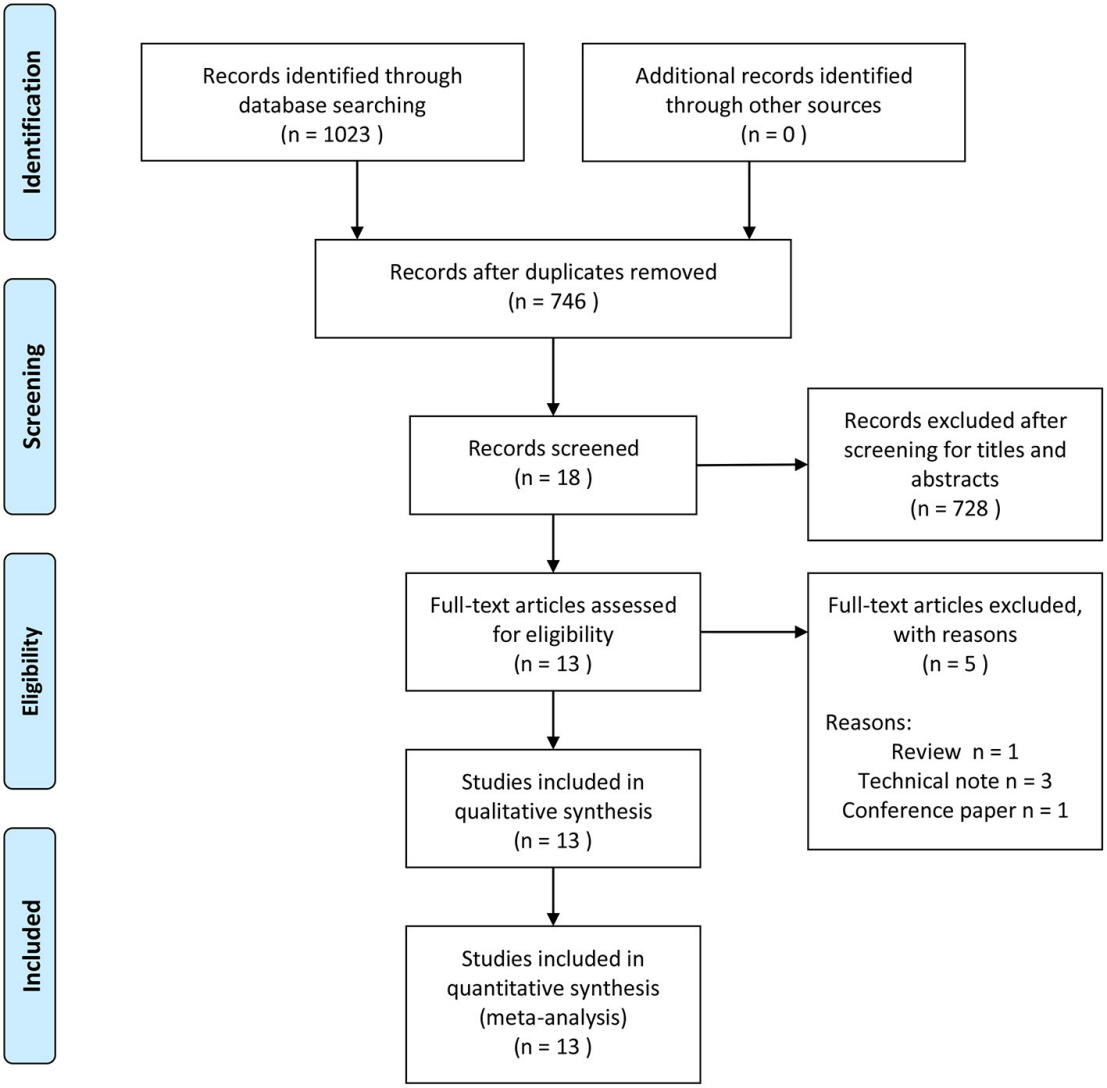
Flowchart for study selection.

We identified 1,023 articles through the database search (Figure 1). After duplicate data removal, 746 studies were remained. After screening of titles and abstracts, 728 studies were excluded. In the final screening, 18 studies underwent full-text review and 5 studies were excluded. Finally, 13 articles were included in the systematic review. These included 1 RCT (34), 1 prospective study (4), and 11 retrospective studies (16, 19, 20, 35–42).

### Characteristics of Included Studies

The systematic review included 13 studies involving 1,005 patients treated with RSA after acute PHF (Table 1). Three studies were conducted in France with French participants, three in Spain, two in Italy, two in the United States, one in Germany, one in Republic of Korea, and one in Switzerland. Among these patients, there were 184 men (18.3%) and 821 women (81.7%), the mean age was 77.5 years (range, 55–89 years), the mean interval between injury and surgery was 7.0 days (range, 0–23 days), and the mean duration of follow-up was 30.2 months (range, 6–90 months). For fracture classification, the Neer classification system (5) was used in 11 studies, the AO/orthopedic trauma association classification system (43) was used in 1 study, and only 1 study did not report the fracture classification they used. For surgical approach, the most commonly used approach was the deltopectoral approach. The other approach included deltoid-splitting, superolateral, deltopectoral, and anterosuperior approach and only one study did not report the approach they used. For the design of prosthesis, five studies only used a fracture-dedicated prosthesis (19, 20, 35, 36, 40) and six studies used a standard prosthesis (4, 16, 34, 37, 39, 42). One study both used a fracture-dedicated prosthesis and a standard prosthesis (41) and one study did not report the specific prosthesis used (38).

**Table 1:** Demographic data of the included studies.

**References**	**Study design**	**Country**	**Gender (No.)**	**Mean Age**	**Fracture type (No.)**	**Approach**	**Implant type**	**Mean Follow-up**
			**M/F**	**Year**				**Month**
Luciani et al. (42)	Retrospective	Italy	5/33	77	3-part (12) 4-part (30)	Deltopectoral	SMR Lima prosthesis	65
Simovitch et al. (19)	Retrospective	United States	17/38	77	OTA/AO type 11-B and C	Deltopectoral	Equinoxe fracture prosthesis	33.7
Reuther et al. (20)	Prospective + Retrospective	Germany	9/72	78.5	4-part Head split Large impression	Deltopectoral Deltoid-splitting	Affinis fracture prosthesis	24.8
Jorge-Mora et al. (41)	Retrospective	Spain	3/55	77	3-part 4-part Head split	Deltopectoral Superolateral	Standardcemented prosthesis, Fracture-dedicated lock prosthesis	26
Boileau et al. (40)	Retrospective	France	4/33	80	3-part (6) 4-part (32)	Superolateral Deltopectoral	Aequalis fracture prosthesis	36
Torrens et al. (39)	Retrospective	Spain	10/31	77.9	3-part (7) 4-part (34)	Anterosuperior	Delta Xtendprosthesis	29
Ohl et al. (38)	Retrospective	France	71/349	77.7	NR	Deltopectoral Deltoid-splitting	Six different prosthesis	28
Chun et al. (37)	Retrospective	Republic of Korea	5/33	80.1	4-part	Deltopectoral	Aequalisprosthesis	37
Grubhofer et al. (36)	Retrospective	Switzerland	6/45	77	3-part (4) 4-part (38) Head split (10)	NR	Zimmer fracture prosthesis	35
Garofalo et al. (35)	Retrospective	Italy	25/62	76.2	3-part 4-part Head split	Deltopectoral	Aequalisfracture prosthesis	27
Sebastia-Forcada et al. (34)	RCT	Spain	4/27	74.7	3-part 4-part	Deltopectoral	SMR Lima prosthesis	29.4
Gallinet et al. (16)	Retrospective	France	14/27	76.9	3-part 4-part	Superolateral	Delta prosthesis, Aequalisprosthesis, Zimmer prosthesis	24
Cuff et al. (4)	Prospective	United States	11/16	74.8	3-part 4-part Head split	Deltopectoral	DJO reverse prosthesis	30

### Quality Assessment

For non-randomized controlled study, we found that all the included studies were considered as high quality (range, 7–9) when using the NOS system. The detailed information of the NOS quality assessment is shown in Supplementary Materials (Supplementary Tables S1, S2). For randomized controlled study, we found that the included study had low risk of bias and the detailed information was in Supplementary Materials (Supplementary Table S3).

### Meta-Analysis for Functional Scores

A total of 11 studies provided detailed information of CS and were included in the meta-analysis (16, 19, 20, 34, 36–42) (Table 2). The overall analysis revealed that the healed greater tuberosity groups had the better CS than the non-healed greater tuberosity groups (MD = 8.44, 95% CI = 4.24 to 12.65, *p* < 0.0001) (Table 3). However, there was high heterogeneity among the included studies (*I*^2^ = 81%, *p* < 0.00001). Four studies provided detailed information of the ASES (4, 19, 20, 37) (Table 2) and there was no significant difference in the ASES between the healed groups and the non-healed groups (MD = 2.62, 95% CI = −1.10 to 6.35, *p* = 0.17) (Table 3). Low heterogeneity existed between the included studies (*I*^2^ = 0%, *p* = 0.90). For the DASH reporting by two studies (16, 42) (Table 2), the healed groups had the lower DASH score than the non-healed groups (MD = −14.14, 95% CI = −18.75 to −9.53, *p* < 0.00001) (Table 3). There was low heterogeneity between the included studies (*I*^2^ = 0%, *p* = 0.38). For the SSV reporting on two studies (36, 38) (Table 2), the healed groups had the higher SSV than the non-healed groups (MD = 12.15, 95% CI = 8.95 to 15.35, *p* < 0.00001) (Table 3). In addition, low heterogeneity existed between the included studies (*I*^2^ = 0%, *p* = 0.46). However, there was no significant difference in the SST (4, 19) (MD = 0.78, 95% CI = −0.16 to 1.73, *p* = 0.10) and the VAS (36, 37) (MD = −0.25, 95% CI = −0.92 to 0.43, *p* = 0.47) scores between the two groups (Table 3). Moreover, there was no significant heterogeneity between the included studies (SSV: *I*^2^ = 0%, *p* = 0.68; VAS: *I*^2^ = 0%, *p* = 0.88).

**Table 2:** Summary of clinical outcomes of the included studies.

**References**	**Group**	**Population**	**Functional scores**	**Range of motion**					
		**(No.)**	**(Mean ± SD)**	**(Mean** **±** **SD, degree)**					
				**Flexion**	**Extension**	**Abduction**	**Adduction**	**External rotation**	**Internal rotation**
Luciani et al. (42)	Healed	23	CS: 72.5 ± 3.82 DASH: 16.8 ± 5.0	135 ± 12.7	NR	119 ± 12.8	NR	28 ± 6.8	NR
	Non-healed	8	CS: 59.6 ± 9.0 DASH: 27 ± 7.8	117 ± 12.8	NR	104 ± 14	NR	12.1 ± 3.1	NR
	Excision	7	CS: 52.2 ± 6.4 DASH: 37.4 ± 7.0	108 ± 10	NR	91 ± 7.2	NR	5.7 ± 1.2	NR
Simovitch et al. (19)	Healed	34	CS: 64.6 ± 12.5 ASES: 79.1 ± 17.2 SPDI: 22.8 ± 25.4 UCLA: 28.3 ± 5.3 SST: 9.2 ± 2.1 VAS: 1.3 ± 2.0 SSF: 7.5 ± 1.9	131.9 ± 32.1	NR	108.3 ± 30	NR	40.0 ± 18.8	3.1 ± 1.5^a^
	Non-healed	21	CS: 63.2 ± 7.4 ASES: 77 ± 14.7 SPDI: 36.2 ± 22.0 UCLA: 28 ± 6.0 SST: 8.2 ± 2.8 VAS: 1.6 ± 1.7 SSF: 7.4 ± 2.1	126.5 ± 32.6	NR	114.1 ± 30.4	NR	28.6 ± 21.5	3.8 ± 1.2^a^
Reuther et al. (20)	Healed^b^	37	CS: 60.3 ± 12.3 ASES: 73.7 ± 14.8	127.6 ± 28.8	29.6 ± 10.2	118.9 ± 30.0	20.9 ± 12.8	19.6 ± 16.4	71.5 ± 20.8
	Partially healed^b^	33	CS: 61.5 ± 13.5 ASES: 78.6 ± 16.4	129.1 ± 35.6	24.1 ± 11.1	120.6 ± 37.6	20.4 ± 11.8	14.2 ± 13.1	73.9 ± 14.8
	Non-healed^b^	11	CS: 62.3 ± 11.7 ASES: 77.6 ± 20.0	134.5 ± 27.8	28.9 ± 8.9	126.8 ± 30.9	19.4 ± 7.3	11.4 ± 16.1	76.4 ± 18.6
Jorge-Mora et al. (41)	Healed	39	CS: 63 ± 10	115 ± 22	NR	115 ± 22	NR	28 ± 8	38 ± 6
	Non-healed	19	CS: 45 ± 8	69 ± 31	NR	68 ± 32	NR	5 ± 7	31 ± 12
Boileau et al. (40)	Healed	32	CS: 64 ± 15 SJSV: 83 ± 15	141 ± 25	NR	NR	NR	27 ± 12	5.2 ± 2.7^a^
	Non-healed	6	CS: 51 ± 12 SJSV: 65 ± 15	115 ± 26	NR	NR	NR	11 ± 12	4.3 ± 1.5^a^
Torrens et al. (39)	Healed	28	CS: 61 ± 9.5	NR	NR	NR	NR	NR	NR
	Non-healed	13	CS: 61 ±11.3	NR	NR	NR	NR	NR	NR
Ohl et al. (38)	Healed	169	CS: 61.0 ± 13.5 SSV: 75.5 ± 14.8	126.7 ± 27.6	NR	NR	NR	22.0 ± 16.2 43.2 ± 26.9 (in 90° of abduction)	4.8 ± 2.7^a^
	Non-healed	131	CS: 54.5 ± 15.2 SSV: 69.1 ± 18.2	113.8 ± 29.9	NR	NR	NR	16.7 ± 20.2 33.0 ± 26.8 (in 90° of abduction)	4.0 ± 2.4^a^
	Excision	120	CS: 53.2 ± 15.2 SSV: 56.5 ± 18.3	100.6 ± 24.9	NR	NR	NR	6.6 ± 6.6 17.5 ± 5.9 (in 90° of abduction)	4.0 ± 2.3^a^
Chun et al. (37)	Healed	14	CS: 67.9 ± 11.6 ASES: 74.3 ± 10.7 VAS: 1.4 ± 1.4	125 ± 18	NR	NR	NR	29 ± 8 25 ± 10 (in 90° of abduction)	15 ± 2^a^
	Non-healed	24	CS: 63.9 ± 8.2 ASES: 70.7 ± 7.2 VAS: 1.6 ± 1.4	127 ± 14	NR	NR	NR	10 ± 9 7 ± 9 (in 90° of abduction)	17 ± 1.0^a^
Grubhofer et al. (36)	Healed	44	CS: 65 ± 14.6 SSV: 86 ± 21.0 VAS: 13.8 ± 1.3	123 ± 28.2	NR	115 ± 36.8	NR	21 ± 18.5	6 ± 4.0^a^
	Non-healed	8	CS: 50 ± 14.6 SSV: 68 ± 21.0 VAS: 12.7 ± 1.3	94 ± 28.2	NR	92 ± 36.8	NR	2 ± 18.5	3 ± 4.0^a^
Garofalo et al. (35)	Healed	66	NR	145.3 ± 19.3	NR	NR	NR	34.3 ± 11.8	45.6 ± 18.9
	Non-healed	21	NR	114.1 ± 15.8	NR	NR	NR	12.9 ± 11.6	25.7 ± 19.1
Sebastia-Forcada et al. (34)	Healed	20	CS: 59.3 ± 10.5	122.1 ± 30.9	NR	116.3 ± 27.4	NR	1.9 ± 1.2^c^	3.1 ± 1.3^a^
	Non-healed	11	CS: 53.9 ± 10.5	117.5 ± 30.9	NR	107.5 ± 27.4	NR	2.5 ± 1.2^c^	2.4 ± 1.3^a^
Gallinet et al. (16)	Healed	18	CS: 65.3 ± 12.7 DASH: 30.1 ± 19.4	127.2 ± 29.4	NR	112.8 ± 26.6	NR	19.7 ± 14.8 49.4 ± 24.5 (in 90° of abduction)	L4 55.6 ± 19.5 (in 90° of abduction)
	Non-healed	23	CS: 50.1 ± 12.7 DASH: 39.3 ± 19.4	96.5 ± 29.4	NR	90.4 ± 26.6	NR	1.6 ± 14.8 10.3 ± 24.5 (in 90° of abduction)	Coccyx 36.8 ± 19.5 (in 90° of abduction)
Cuff et al. (4)	Healed	20	ASES: 78 ± 5.7 SST: 7.7 ± 1.2	147 ± 21.4	NR	NR	NR	28 ± 11.6	50%
	Non-healed	4	ASES: 75 ± 5.7 SST: 7.1 ± 1.2	132 ± 21.4	NR	NR	NR	12 ± 11.6	25%

a *Indicated that the internal rotation was recorded numerically according to the special conversion*.

b *Indicated tuberosity healing categories: healed (loss of < 25% of the initial height of the tuberosity), partially healed (loss of 25–50%), and unhealed (loss of > 50%). c Indicated that the external rotation was recorded numerically according to the special conversion. CS, Constant score; DASH, Disabilities of the Arm, Shoulder and Hand; ASES, American Shoulder and Elbow Surgeons; SPDI, Shoulder Pain and Disability Index; UCLA, University of California at Los Angeles; SST, Simple Shoulder Test; VAS, Visual Analog Scale; SSF, Subjective Shoulder Function; NR, not reported; SJSV, Subjective Shoulder Value*.

**Table 3:** Summary of meta-analyses of the included studies.

					**Heterogeneity**	
**Outcome**	**No. of Studies**	**No. of Patients**	**Effect Estimate**	***P***	**I^**2**^**	**x^**2**^**
CS	11	893	MD = 8.44 (4.24, 12.65)	<0.0001	81%	53.82 (*P* < 0.00001)
ASES	4	198	MD = 2.62 (−1.10, 6.35)	0.17	0%	0.57 (*P* = 0.90)
DASH	2	79	MD = −14.14 (−18.75, −9.53)	<0.00001	0%	0.77 (*P* = 0.38)
SSV	2	472	MD = 12.15 (8.95, 15.35)	<0.00001	0%	0.55 (*P* = 0.46)
SST	2	79	MD = 0.78 (−0.16, 1.73)	0.10	0%	0.17 (*P* = 0.68)
VAS	2	93	MD = −0.25 (−0.92, 0.43)	0.47	0%	0.02 (*P* = 0.88)
Flexion	12	963	MD = 18.77 (10.88, 26.66)	<0.00001	77%	48.68 (*P* < 0.00001)
Abduction	7	356	MD = 15.95 (2.49, 29.41)	0.02	79%	28.57 (*P* < 0.0001)
External rotation	11	932	MD = 16.70 (13.01, 20.38)	<0.00001	73%	36.77 (*P* < 0.0001)
External rotation (in 90° of abduction)	3	499	MD = 21.80 (13.49, 30.10)	<0.00001	72%	7.21 (*P* = 0.03)
Internal rotation	3	226	MD = 8.10 (−3.29, 19.49)	0.16	80%	10.15 (*P* = 0.006)

*CS, Constant score; MD, mean difference; DASH, Disabilities of the Arm, Shoulder and Hand; ASES, American Shoulder and Elbow Surgeons; SPDI, Shoulder Pain and Disability Index; SSV, Subjective Shoulder Value; SST, simple shoulder test; VAS, Visual Analog Scale*.

### Meta-Analysis for ROM

For flexion of the shoulder, 12 studies provided detailed information and were included in the meta-analysis (4, 16, 19, 20, 34–38, 40–42) (Table 2). The healed groups showed significantly improved flexion of the shoulder compared with the non-healed groups (MD = 18.77, 95% CI = 10.88 to 26.66, *p* < 0.00001) (Table 3). However, there was significant heterogeneity among the included studies (*I*^2^ = 77%, *p* < 0.00001). Seven studies reported shoulder abduction (16, 19, 20, 34, 36, 41, 42) and the overall result showed that the healed groups had better abduction than the non-healed groups (MD = 15.95, 95% CI = 2.49 to 29.41, *p* = 0.02) (Table 3). However, high heterogeneity existed among the included studies (*I*^2^ = 79%, *p* < 0.0001). For external rotation of the shoulder, 11 studies provided detailed information (4, 16, 19, 20, 35–38, 40–42) and the healed groups possessed greater external rotation (MD = 16.70, 95% CI = 13.01 to 20.38, *p* < 0.00001) (Table 3). However, significant heterogeneity existed among the included studies (*I*^2^ = 73%, *p* < 0.0001). Three studies reported external rotation of 90° of abduction (16, 37, 38) and the overall analysis revealed that the healed groups had better performance than the non-healed groups (MD = 21.80, 95% CI = 13.49 to 30.10, *p* < 0.00001) (Table 3). However, there was high heterogeneity among the included studies (*I*^2^ = 72%, *p* = 0.03). For internal rotation of the shoulder, three studies provided detailed information (20, 35, 41) and the degree of movement in the healed groups was the same as in the non-healed groups (MD = 8.10, 95% CI = −3.29 to 19.49, *p* = 0.16) (Table 3). However, high heterogeneity existed among the included studies (*I*^2^ = 80%, *p* = 0.006).

### Greater Tuberosity Healing

For fracture-dedicated prosthesis, the mean healing rate of the greater tuberosity was 82.7% (range, 75.9–86%) (Table 4). For a standard prosthesis, the mean healing rate of the greater tuberosity was 63.0% (range, 37–83%) (Table 4). All the included studies described the repair technique of the greater tuberosity included suture fixation and bone graft. In addition, they reported different assessment criterion of tuberosity healing on radiologic assessment. Radiographic evaluation included anteroposterior and lateral views with or without axillary views. Most studies described the healed greater tuberosities as healing of the greater tuberosity in an anatomic position. The greater tuberosities were considered to heal when they were visible and united with the humeral shaft in the anteroposterior view with the shoulder in neutral rotation.

**Table 4:** Summary of healing rates of greater tuberosity and complications between the fracture-dedicated prosthesis and the standard prosthesis.

**Implant type**	**First Author (year)**	**Repaired/Healed greater tuberosity**	**Complication**
		**No. (%)**	
Fracture-dedicated prosthesis	Simovitch et al. (19)	41/34 (83%)	2 Scapular notching 1 Ulnar nerve neuropraxia
	Reuther et al. (20)	81/70 (completely and partially healed, 86%)	7 Scapular notching 1 Periprosthetic fracture 1 Radial nerveparesis 1 Hematoma
	Jorge-Mora et al. (41)	34/26 (76%)	1Prosthesis luxation 1 Infection
	Boileau et al. (40)	38/32 (84%)	1 Hematoma 1 Pulmonary embolism 18 Scapular notching 10 Spur formation
	Grubhofer et al. (36)	48/44 (84.6%)	1 Periprosthetic fracture 1 Hematoma 2 Infection 33 Scapular notching
	Garofalo et al. (35)	87/66 (75.9%)	1 Superficial infection 2 Deep infection 1 Radial nerve neuropraxia 1 Scapular notching
	Total	329/272 (82.7%)	
Standard prosthesis	Luciani et al. (42)	31/23 (74%)	2 Deep infection 2 JointInstability 12 Scapular notching
	Jorge-Mora et al. (41)	24/13 (54%)	1 Periprosthetic fracture
	Torrens et al. (39)	41/28 (68%)	6 Scapular notching 5 Osteophyte 1 Dislocation 6 Transient paresthesia
	Chun et al. (37)	38/14 (37%)	11 Scapular notching
	Sebastia-Forcada et al. (34)	31/20 (64.5%)	1 Deep infection
	Gallinet et al. (16)	27/18 (66.7%)	2 Infection 1 Dislocation 1 Lymphedema 30 Scapular notching
	Cuff et al. (4)	24/20 (83%)	1 Ulnar paresthesia 1 Periprosthetic fracture
	Total	216/136 (63.0%)	

### Complications

For fracture-dedicated prosthesis, postoperative complications included 61 cases of scapular notching, 3 cases of nerve neuropraxia, 2 cases of periprosthetic fractures, 3 cases of hematoma, 1 case of prosthesis luxation, 6 cases of infections, 1 case of pulmonary embolism, and 10 cases of spur formation (Table 4). For a standard prosthesis, there were 59 cases of scapular notching, 5 cases of infections, 2 cases of instabilities, 2 cases of periprosthetic fractures, 5 cases of osteophyte formation, 2 cases of dislocations, 7 cases of paresthesia, and 1 case of lymphedema (Table 4). For complications related to biological factors, there were small differences between the fracture-dedicated prosthesis and the standard prosthesis (6.9 vs. 8.3%, Table 5). However, significant differences were shown in complications between these two types of prostheses, which were related to biomechanical and mechanical factors (Table 5).

**Table 5:** Differences of complication between the fracture-dedicated prosthesis and the standard prosthesis.

**Type of complication**		**Fracture-dedicated prosthesis (*n* = 329)**	**Standard prosthesis (*n* = 216)**
		**No. (%)**	**No. (%)**
Biological	Infection	6 (1.8%)	5 (2.3%)
	Nerve damage	3 (0.9%)	7 (3.2%)
	Spur formation or Osteophyte	10 (3.0%)	5 (2.3%)
	Hematoma or Lymphedema	3 (0.9%)	1 (0.5%)
	Pulmonary embolism	1 (0.3%)	0 (0%)
	Overall	23 (6.9%)	18 (8.3%)
Biomechanical	Periprosthetic fracture	2 (0.6%)	2 (0.9%)
	Scapular notching	61 (18.5%)	59 (27.3%)
	Implant loosening	1 (0.3%)	0 (0%)
	Overall	64 (19.5%)	61 (28.3%)
Mechanical	Dislocation	0 (0%)	4 (1.8%)
Overall		87 (26.4%)	83 (38.4%)

### Sensitivity Analyses

**Figure 2:**
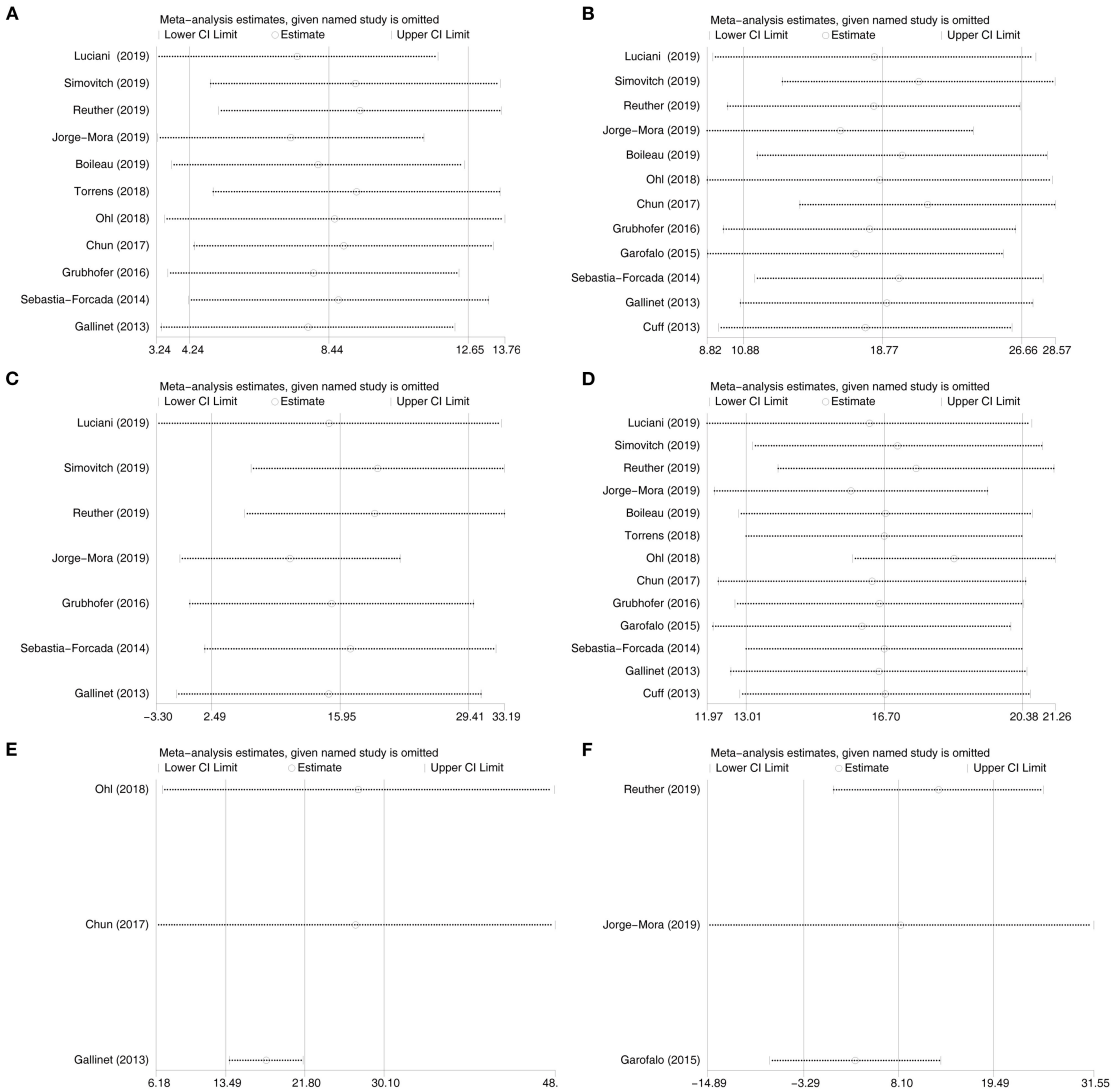
Sensitivity analyses for the Constant score, flexion, abduction, external rotation, external rotation in 90° of abduction. and internal rotation between the healed groups and the non-healed groups. **(A)**: the Constant score, **(B)**: flexion, **(C)**: abduction, **(D)**: external rotation, **(E)**: external rotation in 90° of abduction, and **(F)**: internal rotation.

To assess the reliability of outcomes among included studies, we performed sensitivity analyses by repeating the analysis after removing one study at a time. For the CS and shoulder flexion, the combined estimates did not change markedly with the removal of any one study (Figures 2A,B). For abduction of shoulder, the combined estimates were influenced by studies of Luciani et al. (42), Jorge-Mora et al. (41), and Gallinet et al. (16). After removing the study of Luciani et al., Jorge-Mora et al., or Gallinet et al., there was no significant difference in abduction between the healed groups and the non-healed groups (Figure 2C). For shoulder external rotation and external rotation in 90° of abduction, the combined estimates were stable with the removal of any one study (Figures 2D,E). However, the combined estimates of internal rotation were influenced by the study of Reuther et al. (20). After removing the study of Reuther et al., the shoulder internal rotation was better in the healed groups (Figure 2F).

### Subgroup Analysis

**Figure 3:**
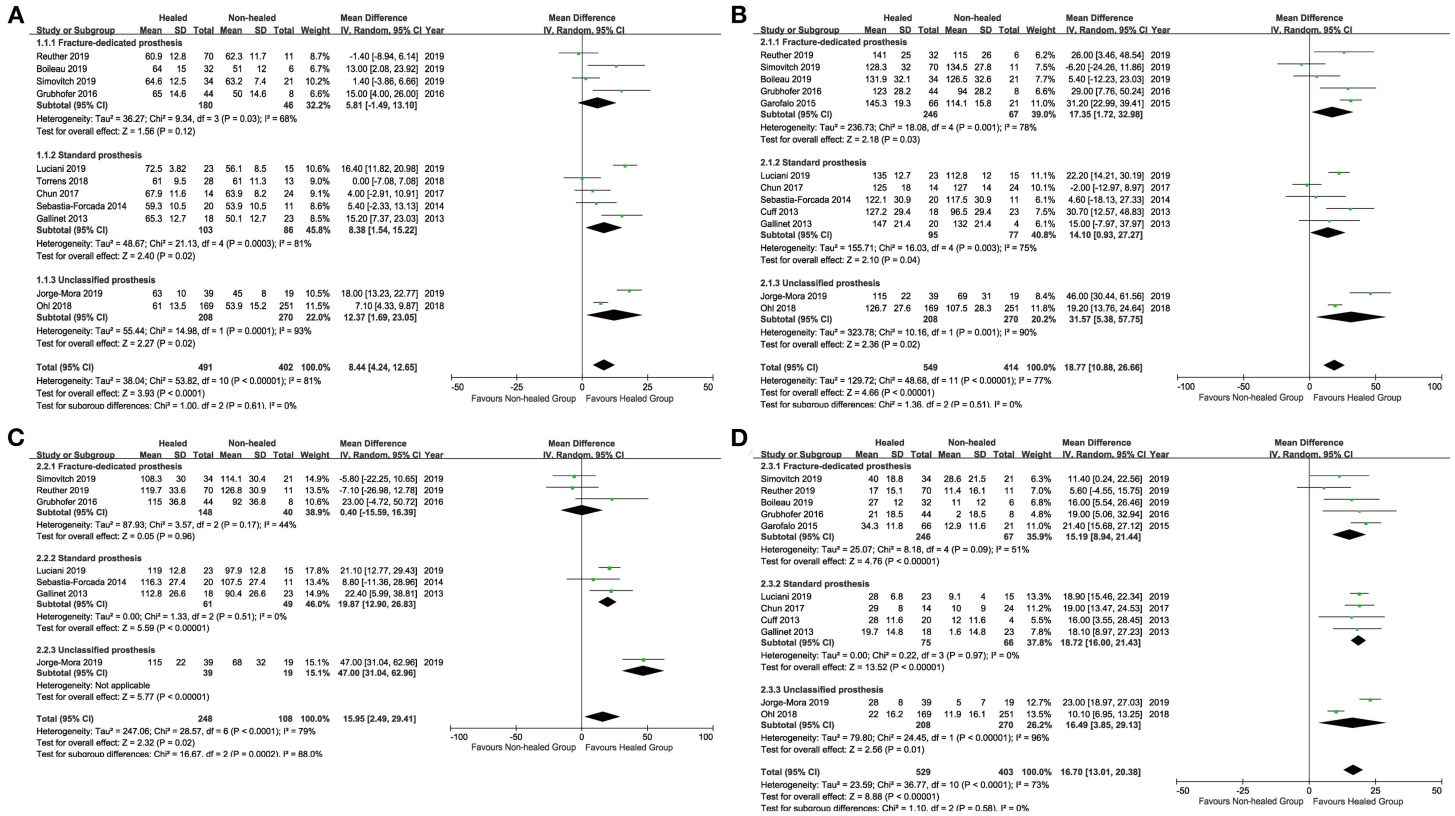
Subgroup analysis for the Constant score, flexion, abduction, and external rotation between the healed groups and the non-healed groups. **(A)**: the Constant score, **(B)**: flexion, **(C)**: abduction, and **(D)**: external rotation.

To explore potential sources of heterogeneity, we further conducted subgroup analysis (Figure 3). Based on the design of prosthesis, we divided the included studies into three subgroups: fracture-dedicated prosthesis, standard prosthesis, and unclassified prosthesis (such as mixed use or not reported). For subgroup analysis of the CS, patients with fracture-dedicated prostheses showed no difference in the CS between the healed groups and the non-healed groups (*p* = 0.12) (Figure 3A). However, patients with standard prostheses showed higher CS in the healed groups (*p* = 0.0003) (Figure 3A). For subgroup analysis of shoulder flexion, the design of prosthesis did not change the overall results (Figure 3B). However, the heterogeneity of the estimates was too high to be reliable. For subgroup analysis of abduction, there was no difference in abduction of patients with fracture-dedicated prostheses between the healed groups and the non-healed groups (*p* = 0.96) (Figure 3C). However, patients with standard prostheses showed better abduction in the healed groups (*p* < 0.00001) (Figure 3C). For subgroup analysis of external rotation, different design of prostheses did not affect the pooled result substantially (Figure 3D).

### Meta-Regression

**Figure 4:**
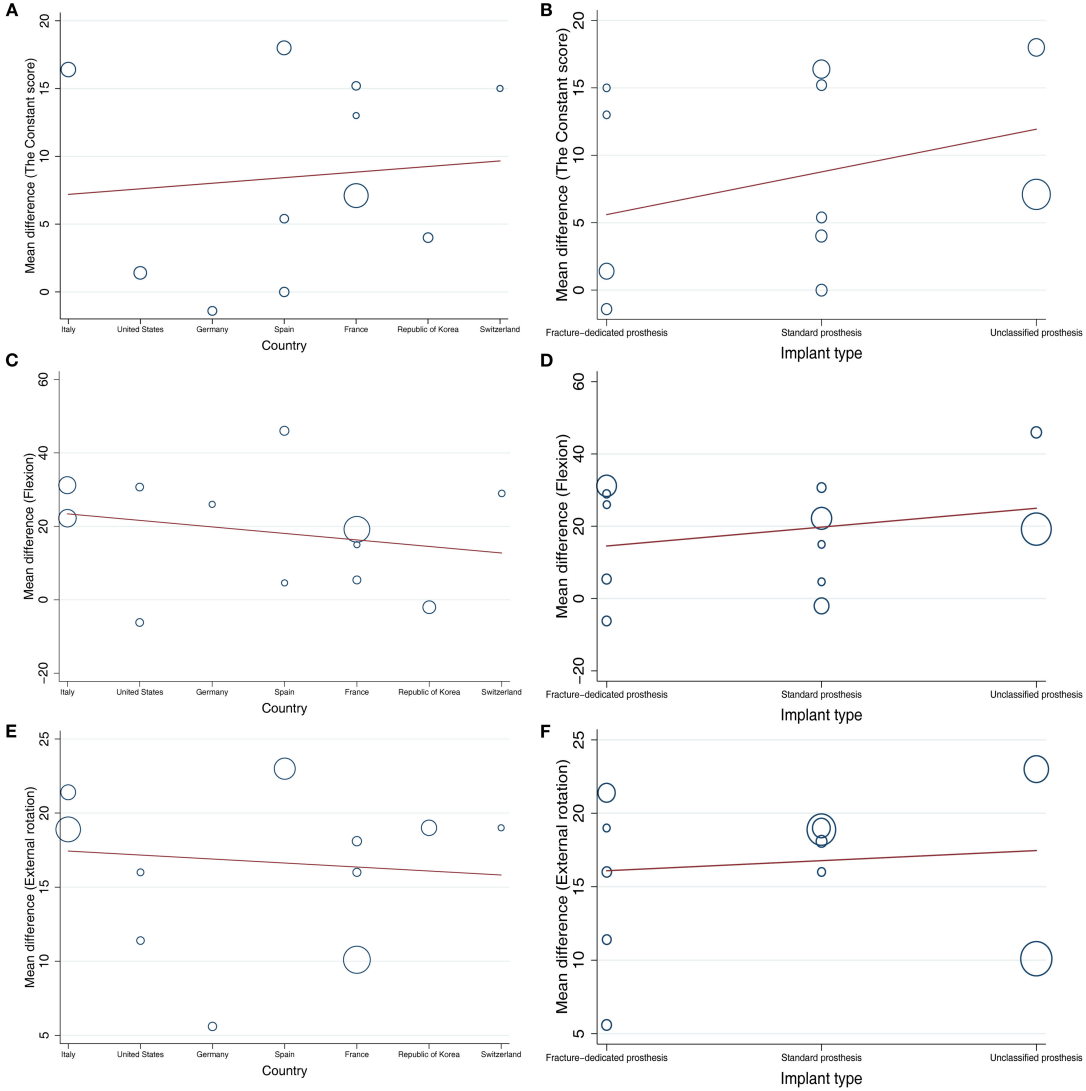
Meta-regression analysis between country or the design of prosthesis and the Constant score, flexion, or external rotation. **(A)**: analysis between country and the Constant score, **(B)**: analysis between the design of prosthesis and the Constant score, **(C)**: analysis between country and flexion, **(D)**: analysis between the design of prosthesis and flexion, **(E)**: analysis between country and external rotation, **(F)**: analysis between the design of prosthesis and external rotation.

To explore potential sources of heterogeneity, we also conducted meta-regression (Figure 4). Country of origin was not associated with the CS (*p* = 0.779) (Figure 4A), shoulder flexion (*p* = 0.185) (Figure 4C), or external rotation (*p* = 0.778) (Figure 4E). In addition, there was no significant relationship between the design of prosthesis and the CS (*p* = 0.312) (Figure 4B), flexion (*p* = 0.422) (Figure 4D), or external rotation (*p* = 0.776) (Figure 4F).

### Publication Bias

**Figure 5:**
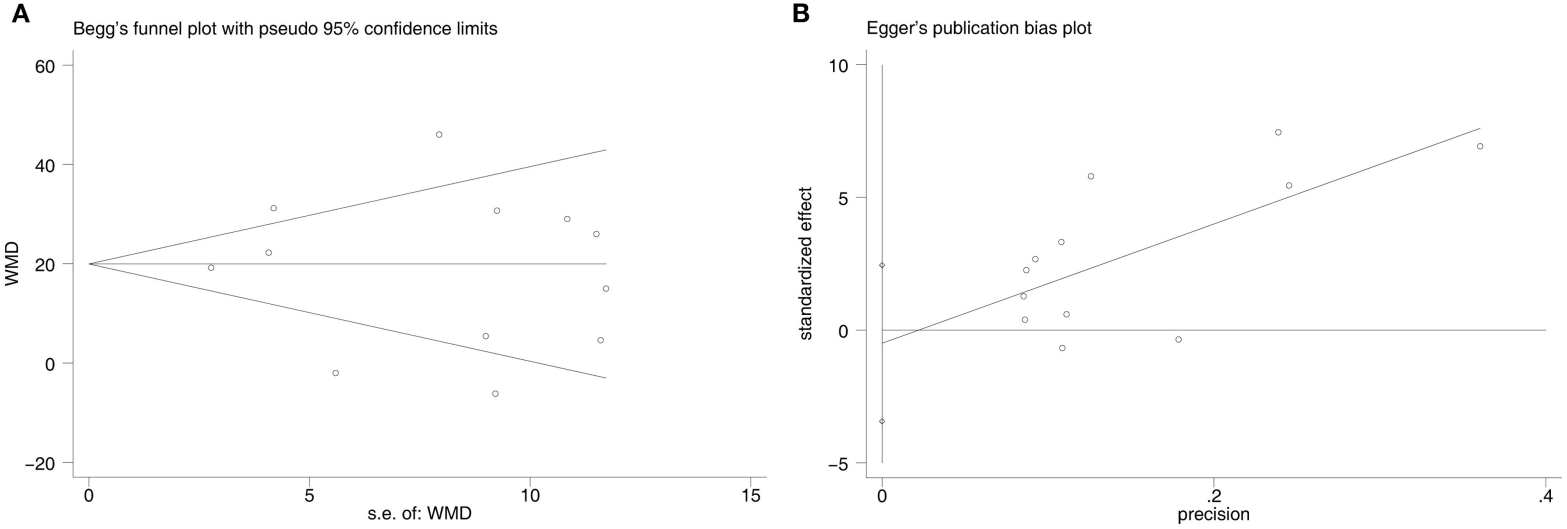
Publication bias of included studies. **(A)**: the Begg's test, **(B)**: the Egger's test.

There was no significant publication bias according to the Begg's test (*p* = 0.732) and the Egger's test (*p* = 0.716) (Figure 5). After performing the trim-and-fill method, the data were unchanged and no study was filled up into the final analysis.

## Discussion

There were several limitations in this study. Firstly, the study was limited by the quality and quantity of the included studies. Secondly, significant heterogeneity in functional scores and the ROM of the shoulder reduced the credibility of the overall results. Quality assessment also showed that there were methodological differences among the included studies. Thirdly, different prostheses were used in the included studies (no detailed information about prosthesis or mixed use of prosthesis) and the impact of the design of the prosthesis on functional scores and the ROM of the shoulder needs further study. Fourthly, inconsistent healing criteria of the greater tuberosity and the validity of subgroup may impact the overall results.

Previous study reported that anatomic healing of the greater tuberosity may result in better functional outcomes and the ROM of shoulder after RSA (18). Our results also confirmed that the healed greater tuberosity groups obtained better clinical outcomes (higher CS, higher SSV, and lower DASH) and better performance in flexion, abduction, external rotation, and external rotation in 90° of abduction. However, a recent finite element analysis about the role of greater tuberosity healing in RSA indicated that greater tuberosity healing did not impact flexion and abduction of shoulder; on the contrary, it did affect the biomechanics of external rotation (44). Interestingly, most studies focus on the advantages of healing of the greater tuberosity and few studies pay attention to the differences of different types of prosthesis, which have an important impact on the healing rate of the greater tuberosity. To the best of our knowledge, this is the first meta-analysis to discuss the effect of the design of prostheses on the healing rate of the greater tuberosity. Besides the design of prosthesis, the use of bone cement and the technique of tuberosity fixation will also affect the healing of the greater tuberosity.

For the design of prosthesis, this study showed that the fracture-dedicated prosthesis gained the higher heading rate of greater tuberosity (82.7%, ranged from 75.9 to 86%) than the standard prosthesis (63.0%, ranged from 37 to 83%). Thus, the design of a prosthesis could affect the healing rate of the greater tuberosity. For design, the benefits of a fracture-dedicated prosthesis may include less proximal metal for better contact of bony tuberosity, proximal coating to promote osseointegration, and medial stem offset that provide more space for tuberosity placement (21). Several studies also advocated the use of dedicated fracture-specific stems during shoulder arthroplasty due to the improvement of greater tuberosity healing (19, 21, 22, 41). Moreover, we found that the design of a prosthesis had an effect on the results of functional scores and the ROM of the shoulder between the healed groups and the non-healed groups. Although the meta-regression did not show the relationship between the design of a prosthesis and functional scores and the ROM, the subgroup analysis gives some useful information. For patients with a fracture-dedicated prosthesis, there was no significant difference in the CS between the healed groups and the non-healed groups. However, for patients with a standard prosthesis, the healed groups produced better CS than the non-healed groups. Unfortunately, the result is dubious due to high heterogeneity between the included studies and the benefit of fracture-dedicated prostheses on functional outcomes needs further study. For abduction, patients with fracture-dedicated prostheses obtained the same degree of motion between the two groups. However, for patients with standard prostheses, the healed groups showed better flexion than the non-healed groups. The result is meaningful due to a low heterogeneity between the included studies. Thus, we are of the opinion that the fracture-dedicated prostheses could improve abduction in patients with a non-healed greater tuberosity.

Although this study did not include the use of bone cement in RSA, the latest meta-analysis showed that bone cement usage was as high as 82.5% (18). The advantages of cementation include the ability to provide good initial stability of implant, a low rate of iatrogenic fracture, the anti-infection ability of antibiotics in the cement, and fixation independent of osteogenesis (45–47). However, the use of cement might inhibit tuberosity healing due to direct thermal reactions, disturbance of local blood flow, and compromise of fixation between fracture fragments (48–50). Singh et al. found that cementation within 5 mm of the greater tuberosity could reduce healing (51). Thus, they reported that bone cement should not be used within 5 mm from the tuberosity fracture or choosing an uncemented prosthesis (51). In addition, the “black-and-tan” technique may reduce the thermal effect of cement in the healing process of the greater tuberosityby using cancellous bone to create an interface between the cement and the tuberosity (48, 52).

In addition to the design of the prosthesis and cementation, this difference in healing rates of the greater tuberosity may be attributed to different fixation techniques. Recent methods to enhance tuberosity healing include suture techniques (53–56) and bone grafting (17, 57, 58). Although there is no consensus on the suture fixation of greater tuberosity, the main fixation method is the combination of vertical fixation and horizontal fixation with/without cerclage fixation. Moreover, horizontal fixation (between tuberosities) is more crucial to tuberosity healing than vertical fixation (prosthesis height) (51, 59). Besides the aforementioned factors, there may be other factors that affect the healing of greater tuberosity such as gender (60).

In conclusion, this study showed that the healed greater tuberosity groups could obtain better the CS, the SSV, and the lower DASH than the non-healed greater tuberosity groups. The healed groups also had better performance in flexion, abduction, external rotation, and external rotation in 90° of abduction than the non-healed groups. In addition, fracture-dedicated prostheses could promote the healing rate of greater tuberosity and it also had an effect on the results of the CS and abduction of the shoulder between the two groups. However, due to limited quantity of high-quality evidence, further rigorous RCTs are needed to confirm the benefits of fracture-dedicated prostheses.

## Data Availability Statement

The original contributions presented in the study are included in the article/Supplementary Material, further inquiries can be directed to the corresponding author/s.

## Author Contributions

S-KH, J-PL, and F-GH contribute to the study design, data analysis, and writing. J-PL and J-HG contribute to the data collections.

## Conflict of Interest

The authors declare that the research was conducted in the absence of any commercial or financial relationships that could be construed as a potential conflict of interest.

## Publisher's Note

All claims expressed in this article are solely those of the authors and do not necessarily represent those of their affiliated organizations, or those of the publisher, the editors and the reviewers. Any product that may be evaluated in this article, or claim that may be made by its manufacturer, is not guaranteed or endorsed by the publisher.
